# Complement C5 activation promotes type 2 diabetic kidney disease via activating STAT3 pathway and disrupting the gut‐kidney axis

**DOI:** 10.1111/jcmm.16157

**Published:** 2020-12-06

**Authors:** Ling Li, Tiantian Wei, Shuyun Liu, Chengshi Wang, Meng Zhao, Yanhuan Feng, Liang Ma, Yanrong Lu, Ping Fu, Jingping Liu

**Affiliations:** ^1^ Kidney Research Laboratory Division of Nephrology and National Clinical Research Center for Geriatrics West China Hospital of Sichuan University Chengdu China; ^2^ Key Laboratory of Transplant Engineering and Immunology Frontiers Science Center for Disease‐Related Molecular Network West China Hospital of Sichuan University Chengdu China

**Keywords:** complement C5, diabetic kidney disease, gut microbiota, inflammation, SCFA, STAT3

## Abstract

Diabetic kidney disease (DKD) is a severe DM complication. While complement C5 up‐regulation and gut dysbiosis are found in T2DM, their roles in DKD are unclear. Here, we investigated the effect of C5 on the gut microbiota during DKD development. Renal C5a/C5a receptor (C5aR) expression changes were measured in T2DM patients and db/db mice. Db/db mice were treated with a C5aR antagonist (C5aRA), and renal function, gut microbiota and renal genome changes were analysed. The effects of C5a and short‐chain fatty acids (SCFAs) on the signal transducer and activator of transcription 3 (STAT3) pathway were examined in vitro. C5a was up‐regulated in glomerular endothelial cells (GECs) of T2DM patients and db/db mice. Although glucose and lipid metabolism were unchanged, C5aR blockade alleviated renal dysfunction, ECM deposition, macrophage infiltration and proinflammatory factor expression in db/db mice. C5aRA partly reversed the declines in gut microbiota diversity and abundance and gut SCFA levels in db/db mice. C5aRA down‐regulated the expression of many immune response‐related genes, such as STAT3, in db/db mouse kidneys. C5aRA and SCFAs suppressed C5a‐induced STAT3 activation in human renal glomerular endothelial cells (HRGECs). Based on our results, C5 hyperactivation promotes DKD by activating STAT3 in GECs and impairing the gut‐kidney axis, suggesting that this hyperactivation is a potential target for the treatment of DKD.

## 1 INTRODUCTION

Type 2 diabetes mellitus (T2DM) is a severe disease characterized by elevated glucose levels due to insulin resistance or inadequate insulin secretion. Diabetic kidney disease (DKD) is one of the most severe microvascular complications of diabetes mellitus (DM) and is the leading cause of end‐stage renal disease (ESRD) and renal failure.^1^ DKD develops in approximately 35% of patients with T2DM^2^ and is strongly associated with increased risks of cardiovascular events and mortality.^3^ The pathological process of DKD is extremely complicated, and multiple factors, such as genetic and epigenetic factors, hyperglycaemia, hypertension and lipid metabolism disorders, have been found to be involved. Moreover, immune system disturbance and inflammation have been considered key regulators in the pathophysiological process of glomerulosclerosis,^4^, ^5^ and there is consensus that the inflammatory response promotes the onset and progression of DKD.

The complement system, an important part of the innate immune system, involves a large group of proteins, including complement components, complement regulatory proteins and complement receptors. The complement system can be activated by pathogens or other stimuli via different pathways, including the antibody‐dependent classical pathway, the alternative pathway or the mannan‐binding lectin pathway. These activated complement pathways start at different points but intersect and lead to a common terminal pathway that eventually produces C5 invertase and converts complement C5 into C5a and C5b. C5a is an important inflammatory mediator that can bind to the G protein‐coupled receptor C5aR and activate its downstream cascade to initiate the inflammatory response. In contrast, the binding of C5a to C5aR is inhibited by a selective, high affinity and competitive antagonist of C5aR (C5aRA), thereby blocking its downstream signalling pathways.^6^ C5a has been reported to stimulate the infiltration of leucocytes into damaged tissues and to induce the release of proinflammatory factors such as interleukin (IL)‐1β, IL‐6 and tumour necrosis factor‐α (TNF‐α). Furthermore, activation of complement C5 is strongly associated with the inflammatory microenvironment of diabetic kidneys, while blockade of C5aR by C5a receptor antagonists prevents endothelial‐mesenchymal transition (EndMT) and alleviates fibrosis in the glomeruli of individuals with DKD.^7^, ^8^ Although the important role of abnormal activation of complement C5 in DKD has been reported, the underlying mechanism is not fully understood.

The gut microbiota is a complex ecosystem, and multiple diseases have been found to be associated with the gut microbiota through intestinal immunity.^9^, ^10^, ^11^ The gut microbiota plays a crucial role in gastrointestinal mucosal permeability and regulates dietary polysaccharide fermentation and absorption, lipid accumulation and the occurrence of T2DM.^12^ Previous studies have found that the gut microbiota is involved in various physiological processes, such as energy metabolism, metabolic signalling and regulation of integrity of the gut barrier.^13^, ^14^, ^15^ Patients with chronic metabolic diseases such as T2DM have been shown to have altered bacterial compositions.^16^ In addition, it has been reported that modification of microbiota composition might affect the outcomes of glomerulopathies.^17^ Recently, we also observed substantial differences in the richness of the gut microbiota and variations in bacterial populations between DKD patients and healthy controls.^18^ However, the effect of complement C5 activation on the gut microbiota in diabetic conditions is not clear.

In this study, we aimed to investigate the effect of complement C5 activation on the gut microbiota during the development of DKD. Changes in complement C5 were examined in T2DM patients and db/db mice. The effects of C5aR blockade on renal function, inflammation, the gut microbiota and short‐chain fatty acid (SCFA) production were evaluated in db/db mice. In addition, the mechanism of the effects of SCFAs on C5a‐induced inflammation was investigated.

## 2 MATERIALS AND METHODS

### 2.1 Human renal biopsy sample collection

All human experiments were conducted in accordance with a study protocol approved by the Ethics Committee of West China Hospital, Sichuan University. Informed consent was obtained from all patients according to the Declaration of Helsinki. Renal tissues were obtained from renal biopsies of Type 2 DKD patients. Normal renal tissues obtained from surgical nephrectomy of renal carcinoma patients were used as controls.

### 2.2 Animal experiment

All animal experiments were approved by the Animal Care and Use Committee of Sichuan University and were conducted in accordance with the National Institutes of Health Guide for the Care and Use of Laboratory Animals. Db/m mice (25‐30 g) and db/db mice (45‐55 g) were purchased from GemPharmatech Co., Ltd. The animals were housed in standardized conditions under constant temperature and humidity and a 12‐hour light/dark cycle and were fed sterilized food and given access to distilled water ad libitum. The mice were randomly divided into 3 groups (8 per group): the control group (db/m mice), the DKD group (db/db mice) and the DKD + C5aR antagonist (C5aRA) group (db/db mice + C5aRA). C5aRA (Cat number: HY‐16992A, lot number: 23314) was purchased from MedChemExpress LLC, and its dose used in vivo was chosen based on the previous reports with slight modification.^8^, ^19^ The mice in the DKD + C5aRA group were administered 0.5 mg/kg/d C5aRA (dissolved in DMSO/PBS, v/v = 1:100) by gavage, and the mice in the control and DKD groups were treated with equal amounts of vehicle (DMSO/PBS, v/v = 1:100). The interventions were started in mice at 10 weeks of age. After 4 weeks of treatment, the mice were sacrificed by overdose of 1% pentobarbital sodium, and serum, urine, faecal and renal samples were collected for further analysis.

### 2.3 Biochemical measurement

Biochemical analysis of serum was performed on an Automatic Biochemistry Analyzer (Cobas Integra 400 Plus, Roche) with commercial kits. The following parameters were assessed: glucose, cholesterol (CHOL), triglyceride (TGs), low‐density lipoprotein (LDL), serum creatinine (Scr), blood urea nitrogen (BUN), uric acid (UA), urine microalbumin and urine creatinine. The urinary albumin‐to‐creatinine ratio (UACR) was calculated.

### 2.4 Immunofluorescence (IF) staining

Frozen sections of kidneys from patients or mice were fixed with 10% formalin and incubated with rabbit anti‐C5aR (Abcam), rabbit anti‐C5a (Abcam), rabbit anti‐p‐signal transducer and activator of transcription (STAT) 3 (Abclonal), mouse anti‐p‐STAT3 (Santa Cruz) and mouse anti‐CD31 (Santa Cruz) antibodies at 4°C overnight. After washing with PBS, the sections were incubated with diluted fluorescence‐conjugated secondary antibodies, including goat anti‐rabbit IgG/TRITC (Jackson ImmunoResearch) and goat antimouse IgG/FITC (Jackson ImmunoResearch), at 37°C in the dark for 1 hour and then stained with DAPI (Calbiochem). Micrographs of the stained sections were acquired on a confocal laser scanning microscope (N‐STORM & A1, Nikon Tokyo). Morphologic analysis of the images was performed with ImageJ software (NIH).

### 2.5 Measurement of H_2_O_2_ concentrations

The concentrations of H_2_O_2_ in serum samples from mice were measured by using a commercial Hydrogen Peroxide Assay Kit (Beyotime Biotechnology) according to the manufacturer's instructions.

### 2.6 ELISA of C5a concentrations

The C5a concentrations in mouse serum and renal tissue were assayed by using a commercial ELISA detection kit (Mouse Complement C5a ELISA Kit, ab193718, Abcam) according to the manufacturer's instructions.

### 2.7 Serum cytokine assay

The levels of serum cytokines including IL‐6 and MCP‐1 were analysed using a Mouse Premixed Multi‐Analyte Kit (R&D Systems) according to the manufacturer's instructions. In brief, 50 µL of sample or standard was added to each well of a 96‐well plate and mixed with 50 µL of microparticle cocktail. The plate was incubated at room temperature for 1 hour. After repeated washing, each sample was incubated with 50 µL of biotin‐antibody cocktail and then with 50 µL of streptavidin‐PE at room temperature for 1 hour. After washing, samples and standards were read on a Luminex 200 analyzer.

### 2.8 16S ribosomal ribonucleic acid (rRNA) sequencing of the gut microbiota

Mouse faecal samples were analysed by 16S rRNA sequencing analysis. Briefly, DNA was extracted from the samples by using an EZNA Soil DNA Kit (Omega Bio‐tek) according to the manufacturer's protocols. The V3‐V4 hypervariable regions of the bacterial 16S rRNA gene were amplified with the primers 338F (5′‐ACTCCTACGGGAGGCAGCAG‐3′) and 806R (5′‐GGACTACHVGGGTWTCTAAT‐3′) in a thermocycler PCR system (GeneAmp 9700, ABI). The PCR products were extracted from a 2% agarose gel and purified using an AxyPrep DNA Gel Extraction Kit (Axygen Biosciences). The purified amplicons were pooled in equimolar amounts and subjected to paired‐end sequencing on an Illumina MiSeq platform (Illumina) according to standard protocols by Majorbio Bio‐Pharm Technology Co., Ltd. The resulting data were analysed on the free online Majorbio Cloud Platform (www.majorbio.com).

### 2.9 SCFA analysis

SCFAs were extracted from faecal samples (100 mg) or serum samples (100 µL) using methanol (500 µL, Sigma). After centrifugation (14 000 rpm at 4°C for 10 minutes), the supernatant of the samples was collected and incubated with Na_2_SO_4_ (10 mg, Sigma) to remove water. For derivatization, 500 µL of propanol/pyridine mixture (v/v = 3:2, Sigma) and 100 µL of propyl chloroformate (PCF, Sigma) were added to the samples. After the reaction, the samples were extracted with hexane (500 µL) following centrifugation, and then, the derivatives of SCFAs were assessed. The samples were analysed on an Agilent 7890B gas chromatography (GC) system coupled to an Agilent 5977A MSD system (Agilent Technologies Inc) with an HP‐5 column (30 m × 0.25 mm × 0.25 µm; Agilent J&W Scientific). Helium (>99.999%) was used as a carrier gas at a constant flow rate of 1 mL/min. The temperature of the inlet was set to 260°C. One microlitre of sample was injected in split mode at a ratio of 10:1, and the solvent delay time was 2.2 minutes. The oven temperature was initially held at 80°C for 2 minutes, ramped to 200°C at a rate of 10°C/min and finally held at 250°C for 3 minutes. The temperature of the electron impact (EI) ion source and the quadruple were set to 230°C and 150°C, respectively. The electron energy was 70 eV, and the mass spectrum data were collected in scan mode (m/z 30‐600). The concentrations of SCFAs were calculated by using a mixed standard solution.

### 2.10 Transcriptome RNA sequencing (RNAseq) analysis

Total RNA was extracted from renal tissues using TRIzol reagent (Invitrogen Waltham), and genomic DNA was removed using DNase I (TaKara Bio Inc). Then, RNA quality was determined with a 2100 Bioanalyzer (Agilent) and quantified using an ND‐2000 (NanoDrop Technologies). The RNAseq transcriptome library was prepared with a TruSeq^TM^ RNA sample preparation kit from Illumina according to the manufacturer's instructions using 1 µg of total RNA. Briefly, RNA was isolated according to the polyA selection method with oligo (dT) beads and then fragmented in fragmentation buffer. cDNA was synthesized using a SuperScript cDNA Synthesis Kit (Invitrogen) with random hexamer primers (Illumina). Then, the synthesized cDNA was subjected to end repair, phosphorylation and ‘A’ base addition according to Illumina's library construction protocol. The libraries were size‐selected for cDNA target fragments of 200‐300 bp on 2% agarose gels and then subjected to PCR amplification using Phusion DNA polymerase (NEB) for 15 PCR cycles. After quantification with a TBS380 instrument, the paired‐end RNAseq sequencing libraries were sequenced with an Illumina HiSeq X Ten System. The data were analysed with the free online Majorbio Cloud Platform (www.majorbio.com).

### 2.11 Cell culture and treatment

Human renal glomerular endothelial cells (HRGECs) were purchased from ScienCell Research Laboratories (ScienCell) and cultured in endothelial cell medium (ScienCell) containing 5% foetal bovine serum (FBS) and 1% endothelial cell growth supplement (ECGS) at 37°C in a 5% CO_2_ atmosphere. The cells were pretreated with SCFAs, including acetate (20 mmol/L, Shanghai Macklin Biochemical Co., Ltd.), propionate (10 mmol/L, Macklin) and butyrate (5 mmol/L, Macklin), or C5aRA (5 µmol/L, MedChemExpress) for 1 hour as previously described.^20^, ^21^ Afterwards, cells were stimulated with recombinant human C5a (50 nmol/L, Sino Biological Inc).

### 2.12 Western blot analysis

Cells were lysed in radioimmunoprecipitation assay (RIPA) buffer on ice with protease and phosphatase inhibitors (Calbiochem). The total protein concentration was determined with a BCA Protein Assay Kit (CWBIO). Equal amounts of protein were separated by 12% sodium dodecyl sulphate‐polyacrylamide gel electrophoresis (SDS‐PAGE) and then transferred to polyvinylidene difluoride (PVDF) membranes (Merck Millipore). The membranes were blocked with TBST containing 5% non‐fat milk and incubated with primary antibodies against C5a (Abcam), C5aR (Abcam), IL‐1β (Abclonal), MCP‐1 (Abcam), STAT3 (Abclonal), p‐STAT3 (Abclonal) and β‐actin (Santa Cruz) overnight at 4°C. After washes with TBST, the PVDF membranes were incubated with horseradish peroxidase (HRP)‐conjugated secondary antibodies (Santa Cruz) at 37°C for 1 hour. The protein bands on the PVDF membranes were visualized with an enhanced chemiluminescence kit (Millipore) and quantified by NIH ImageJ software, and the protein levels were normalized to those of β‐actin.

### 2.13 Transmission electron microscopy (TEM) assay

Renal tissues from mice were fixed with a 2.5% glutaraldehyde solution and then dehydrated and embedded in EPON resin. Ultra‐thin sections of embedded tissues were stained with a 5% uranyl acetate and lead citrate solution and observed using a TEM (JEM‐1400PLUS, JEOL Ltd.).

### 2.14 Histological examination

Renal tissues fixed in 10% formalin were embedded in paraffin, cut into 4 µm sections and stained with haematoxylin and eosin (HE) and periodic acid‐Schiff (PAS). For immunohistochemical (IHC) staining, renal sections were incubated with primary antibodies against 8‐hydroxydeoxyguanosine (8‐OHdG; Abcam), F4/80 (HuaBio, Hangzhou, China), IL‐1β (HuaBio), MCP‐1 (Abcam) and Claudin‐1(ZenBio) overnight at 4°C and then incubated with HRP‐conjugated secondary antibodies (Millipore) and 3,3‐diaminobenzidine (DAB) substrate. Micrographs of the stained sections were captured by light microscopy (Zeiss Imager A2), and the staining was quantified by ImageJ (NIH).

### 2.15 Statistical analysis

All data are presented as the mean ± SD or as the median (Q1‐Q3) and were analysed by SPSS software (version 11.5, IBM Corporation). Comparisons among groups were analysed with one‐way analysis of variance (ANOVA) and Tukey's post hoc test, and *P* < .05 was considered to indicate a significant difference.

## 3 RESULTS

### 3.1 Increased complement C5 activation in the kidneys of DKD patients and db/db mice

**FIGURE 1:**
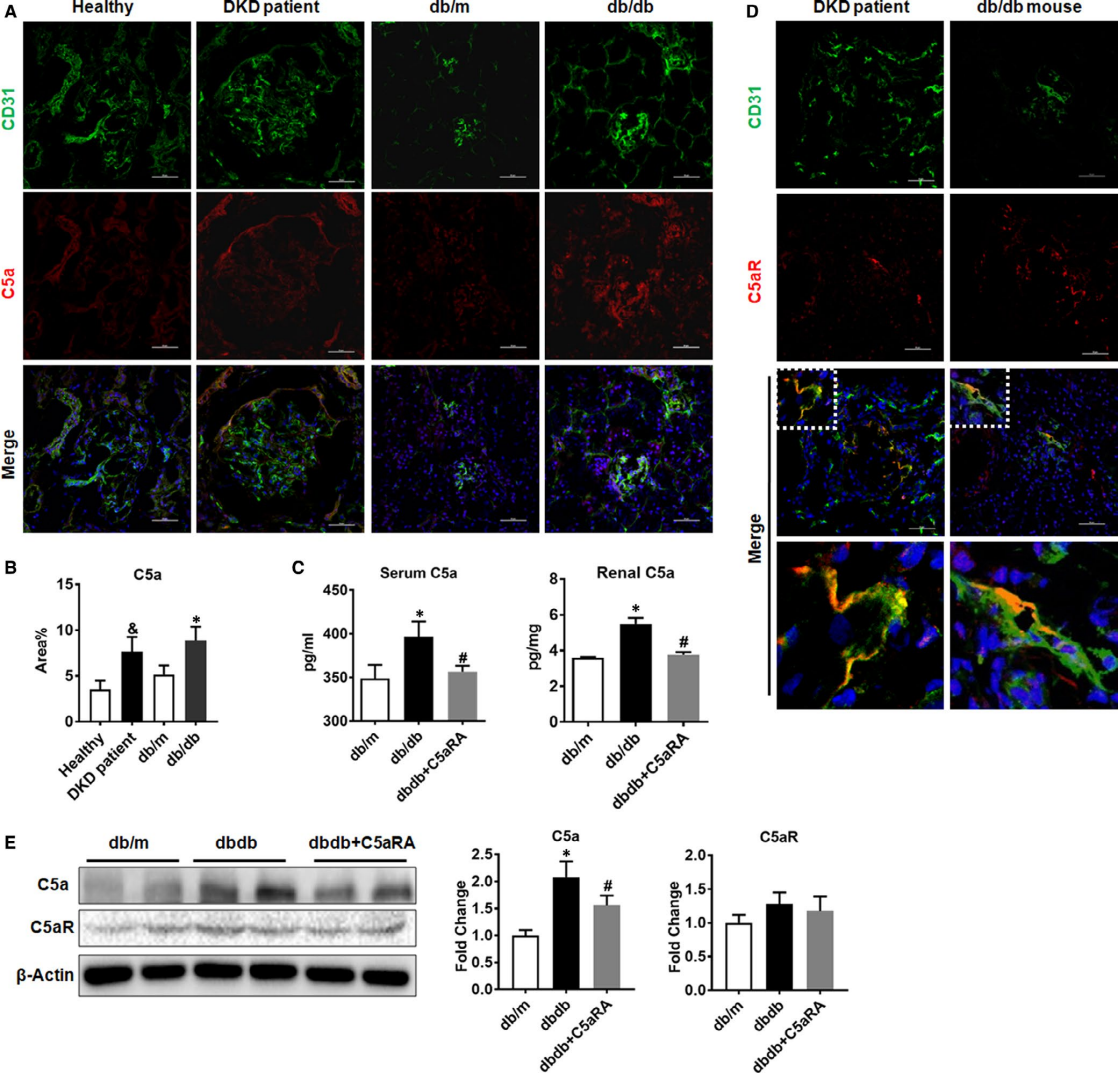
Increased complement C5 activation in kidneys of DKD patients and db/db mice. (A) Double‐IF staining of C5a and CD31 in the kidneys of humans and mice (scale bar = 50 µm). (B) The fluorescent intensity of C5a in kidney detected by IF staining. (C) The concentration of C5a in serum and renal tissue of mice measured by ELISA. (D) Double‐IF staining of C5aR and CD31 in the kidneys of humans and mice (scale bar = 50 µm). (E) The protein level of C5a and C5aR in kidney of mice measured by Western blot. ^&^ *P* < .05, DKD patient vs Healthy. *P < .05, db/db or db/db + C5aRA group vs db/m; ^#^ *P* < .05, db/db + C5aRA group vs db/db group

The coexpression of C5a and CD31 in the kidneys of humans and mice was observed. DKD patients and db/db mice showed higher levels of renal C5a than healthy controls and db/m mice, respectively (Figure 1A,B). The ELISA results confirmed that C5a levels were increased in the serum and kidneys of db/db mice but were reduced by C5aRA treatment (Figure 1C). C5aR was also expressed in CD31‐positive glomerular endothelial cells (GECs) in kidneys from DKD patients and db/db mice (Figure 1D), but there were no significant differences in C5aR expression between db/m and db/db mice or between healthy controls and DKD patients (Figure S1). The Western blot results confirmed that db/db mice had higher levels of renal C5a than db/m mice, while C5a levels were reduced in the kidneys of db/db mice treated with C5aRA. However, there were no significant differences in C5aR levels among the three groups (Figure 1E).

### 3.2 Effects of C5aR blockade on general and renal function parameters in db/db mice

**FIGURE 2:**
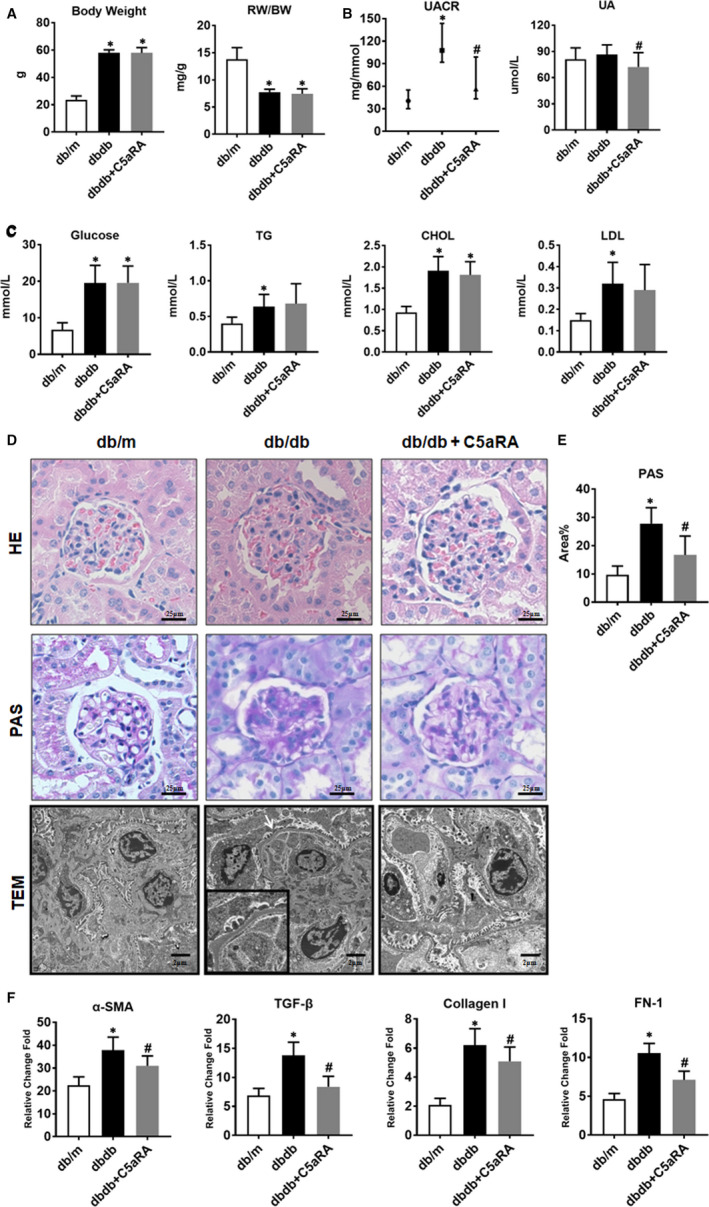
Effect of C5aR blockade on general and renal function parameters in db/db mice. (A) Measurement of bodyweight (BW) and renal weight/bodyweight (RW/BW) in mice. (B) Measurement of UACR and UA in mice. (C) Measurement of glucose and lipids (CHOL, TG and LDL) in mice. (D) Representative images of H&E, PAS staining (scale bar = 25 µm) and TEM (scale bar = 2 µm) in renal tissues from mice. (E) Qualitative analysis of PAS‐positive area in renal sections from mice. (F) The gene expression of α‐SMA, TGF‐β, collagen I and FN‐1 in kidneys of db/db mice detected by RNAseq analysis. *P < .05, db/db or db/db + C5aRA group vs db/m; ^#^ *P* < .05, db/db + C5aRA group vs db/db group

The db/db mice exhibited a higher bodyweight (BW), glucose levels and lipid (CHOL, TG and LDL) levels and lower renal weight (RW)/BW ratios than the db/m mice. However, there were no significant differences in these parameters between the db/db mice and the db/db mice treated with C5aRA (Figure 2A,C). The UA levels and UACR were higher in db/db mice than in db/m mice, but the elevations in UA and UACR were suppressed by C5aRA treatment (Figure 2B). However, there were no significant differences in BUN or sCr among the three groups (Figure S2). Moreover, according to the HE, PAS and TEM results, db/db mice showed renal injury, including tubular hypertrophy, mesangial proliferation, podocyte foot process fusion and basement membrane thickening, while these renal lesions were partially attenuated by the C5aRA treatment (Figure 2D,E). In addition, C5aR blockade suppressed the up‐regulation of profibrotic genes such as α‐SMA, TGF‐β, collagen I and FN‐1 in the kidneys of db/db mice (Figure 2F).

### 3.3 Effects of C5aR blockade on oxidative stress and inflammation in db/db mice

**FIGURE 3:**
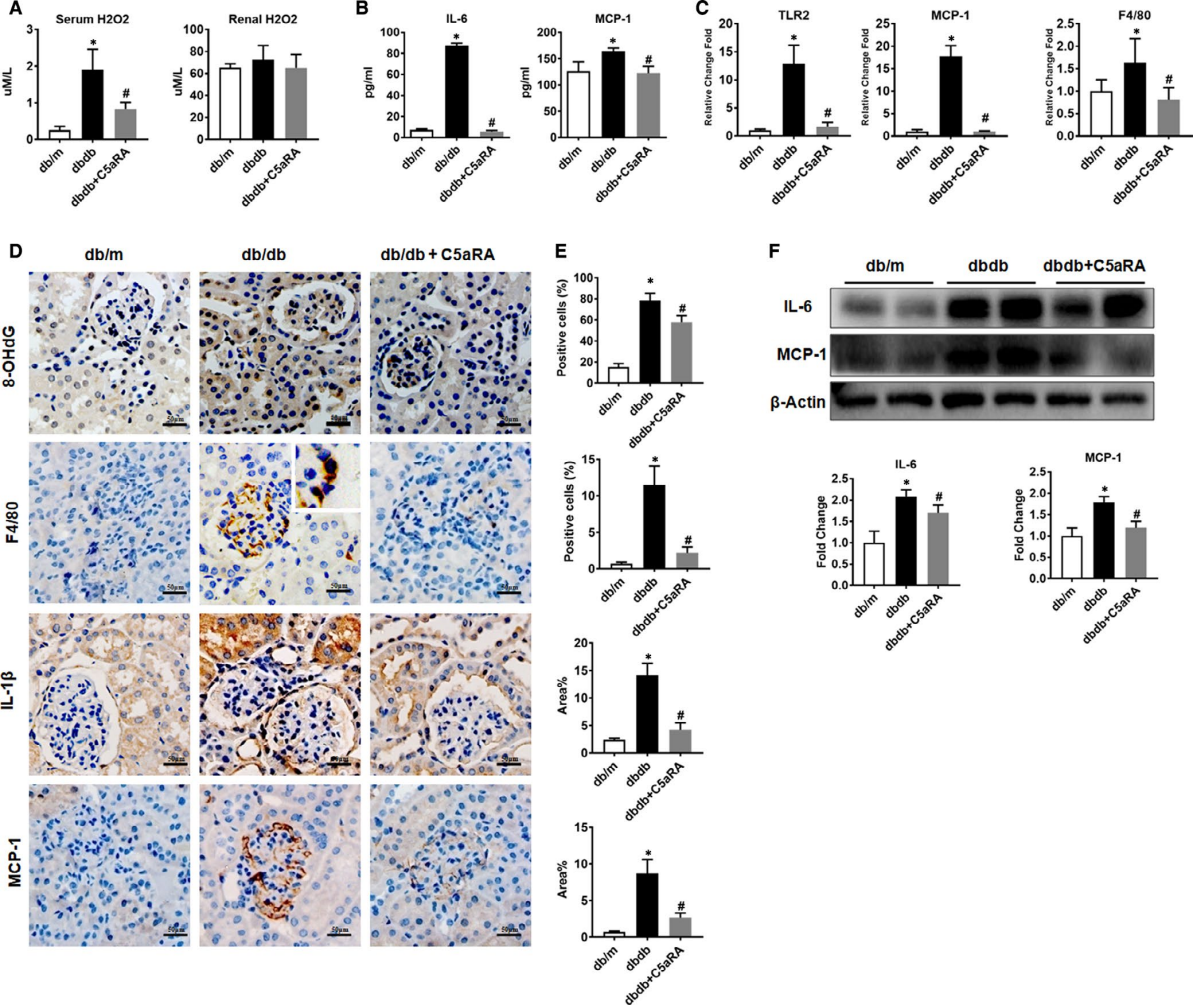
Effect of C5aR blockade on oxidative stress and inflammation in db/db mice. (A) Measurement of H_2_O_2_ in serum and renal tissue of mice by commercial kit. (B) Measurement of IL‐6 and MCP‐1 in serum of mice by Luminex assay. (C) The gene expression of TLR2, MCP‐1 and F4/80 in kidneys of mice detected by RNAseq analysis. (D‐E) Measurement of 8‐OHdG, F4/80‐positive macrophages, IL‐1β and MCP‐1 expression in the kidneys of mice by IHC staining (scale bar = 50 µm). (F) Measurement of IL‐6 and MCP‐1 protein expression in the kidney of mice by Western blotting. *P < .05, db/db or db/db + C5aRA group vs db/m; ^#^ *P* < .05, db/db + C5aRA group vs db/db group

Compared with db/m mice, db/db mice showed higher serum levels of H_2_O_2_, but the elevations in db/db mice were significantly reduced after C5aRA treatment (Figure 3A). There were no significant differences in renal H_2_O_2_ levels among the three groups. The level of 8‐OHdG, a biomarker of oxidative stress,^22^, ^23^ was increased in the kidneys of db/db mice compared to control mice, while it was reduced to some extent by the C5aRA treatment. In addition, C5aR blockade reduced the levels of proinflammatory cytokines (IL‐6 and MCP‐1) in serum (Figure 3B) and reduced the expression of proinflammatory genes such as TLR2, MCP‐1 and F4/80 in the kidneys of db/db mice (Figure 3C). According to the IHC staining, the C5aRA treatment reduced the glomerular F4/80‐positive macrophage infiltration and proinflammatory factor (IL‐1β and MCP‐1) expression in the kidneys of db/db mice (Figure 3D,E). Furthermore, the protein expression of cytokines including IL‐6 and MCP‐1 was lower in the kidneys of db/db mice with C5aR blockade than in those of db/m mice (Figure 3F).

### 3.4 Effect of C5aR blockade on gut microbiota homeostasis in db/db mice

**FIGURE 4:**
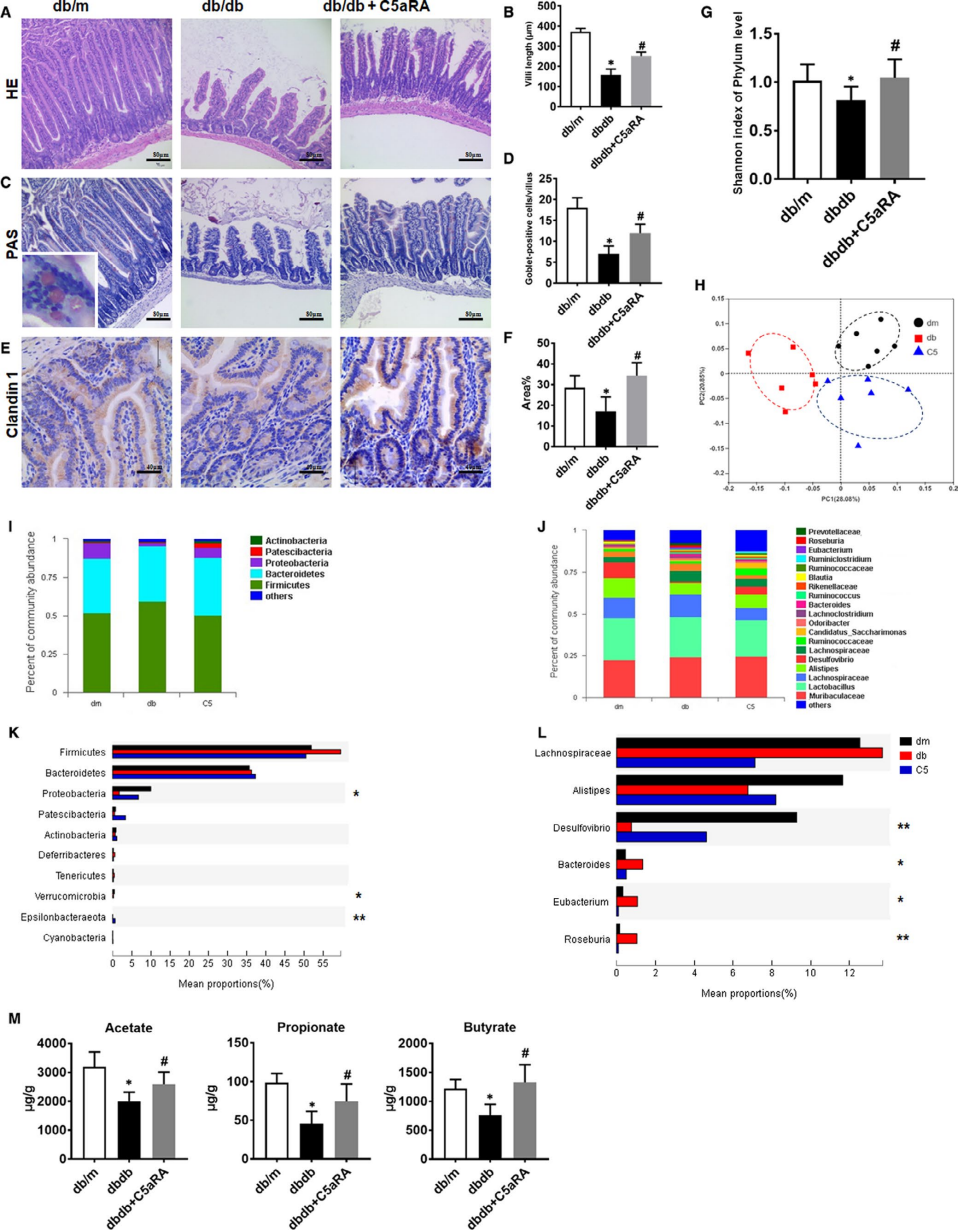
Effect of C5aR blockade on gut microbiota homeostasis in db/db mice. (A and C) Representative images of H&E and PAS staining of colons from mice (scale bar = 80 µm). (B and D) Quantification of the villi length (µm) and goblet‐positive cells detected by H&E and PAS staining. (E and F) Measurement of Claudin 1 expression in the small intestine of mice by IHC staining (scale bar = 40 µm). (G) The Shannon index of gut microbiota in mice detected by 16S rRNA sequencing. (H) Principal co‐ordinates analysis (PCoA) of gut microbiota in mice using 16S rRNA sequencing. (I and K) Measurement of the gut microbiota abundance of mice at the phylum level. (J and L) Measurement of the gut microbiota abundance of mice at the genus level. (M) GC‐MS analysis of SCFAs (acetate, butyrate and propionate) level in faecal samples of mice.*P < .05, db/db or db/db + C5aRA group vs db/m group; ^#^ *P* < .05, db/db + C5aRA group vs db/db group

Compared with db/m mice, db/db mice displayed shorter villus lengths (as indicated by HE staining), fewer mucin‐positive goblet cells (as indicated by PAS staining) and a lower level of claudin‐1 (tight junction protein) expression in the small intestine, whereas C5aRA prevented the reductions in villus length, goblet cell numbers and levels of the claudin‐1 protein in the small intestine of db/db mice (Figure 4A‐F). In addition, db/db mice showed a higher level of intestinal C5a than db/m mice, while intestinal C5a deposition was further decreased by the C5aRA treatment (Figure S3). Moreover, C5aRA reversed the reductions in gut microbiota diversity (as determined by the Shannon index) in db/db mice (Figure 4G). However, the richness of the gut microbiota, as indicated by the Sobs, Chao and abundance‐based coverage estimator (ACE) indexes, was not affected by diabetes or C5aRA (Figure S4). The principal coordinate analysis (PCoA) revealed a significant difference in the microbiota composition among the three groups at the operational taxonomic unit (OTU) level (Figure 4H) using the jaccard distance, and the *P* value was ~.001 according to the PERMANOVA. Microbe abundance was also analysed. At the phylum level, the abundances of Proteobacteria, Verrucomicrobia and Epsilonbacteraeota were significantly reduced in the db/db group compared to the db/m group, while C5aRA restored the levels of Proteobacteria and Epsilonbacteraeota in db/db mice (Figure 4I,J). At the genus level, the abundance of Desulfovibrio was significantly reduced in the db/db group, while those of Bacteroides, Eubacterium and Roseburia were significantly increased; however, these changes were reversed by C5aRA treatment (Figure 4K,L). As a result of these changes, SCFA production by the gut microbiota was disrupted by the diabetic status; faecal samples from db/db mice contained lower levels of acetate, butyrate, propionate, pentanoic acid and isobutanoic acid than faecal samples from db/m mice. However, C5aRA treatment partly restored the levels of acetate, butyrate and propionate in db/db mice (Figure 4M). There were no significant differences in the levels of the other SCFAs among the three groups (Figure S5A). In addition, lower levels of acetate, propionate and hexanoic acid were detected in the serum of db/db mice than in the db/m group, and their levels were restored by the C5aRA treatment (Figure S5B). However, significant differences in the levels of the other SCFAs were not observed between groups, and the possible explanation may be their low levels in serum.

### 3.5 Effect of C5aR blockade on the renal transcriptome in db/db mice

**FIGURE 5:**
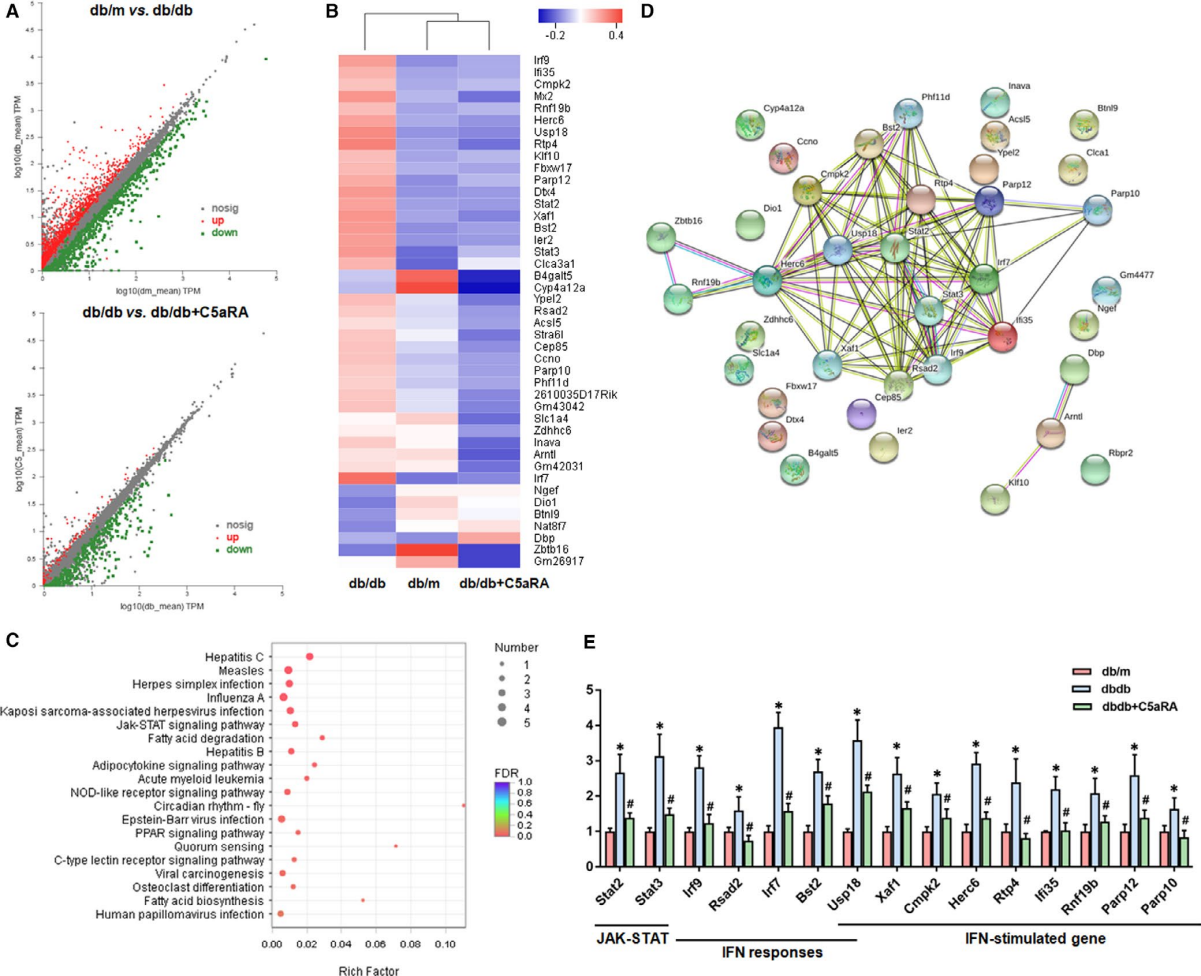
Effect of C5aR blockade on renal transcriptome in db/db mice. (A) Scatter plot of renal gene expression between different groups of mice detected by RNAseq analysis. (B) Hierarchical clustering heatmap analysis of the changed genes among the three groups. (C) KEGG pathway enrichment analysis of the changed genes in kidneys of mice. (D) Gene network analysis of the changed genes in kidneys of mice. (E) The expression of the changed genes in kidneys of mice detected by RNAseq analysis. *P < .05, db/db or db/db + C5aRA group vs db/m group; ^#^ *P* < .05, db/db + C5aRA group vs db/db group

To reveal the protective mechanism of C5aR blockade on the kidneys, RNAseq analysis of renal tissues was performed. As shown in Figure 5A,B, diabetes caused dysregulation of many genes in the kidneys of db/db mice, but the alterations in the gene expression profile were partly reversed by C5aRA treatment. Furthermore, Kyoto Encyclopedia of Genes and Genomes (KEGG) enrichment analysis and gene network analysis revealed that immune response‐related inflammation was one of the leading biological processes affected by C5aR blockade (Figure 5C,D). In brief, genes involved in the JAK‐STAT pathway, including Stat2 and Stat3; type I interferon (IFN) response genes, including Irf9, Rsad2, Usp18, Irf7 and Bst2; and IFN‐stimulated genes (ISGs), including Xaf1, Usp18, Cmpk2, Herc6, Rtp4, Ifi35, Rnf19b and Parp10/12, were up‐regulated in the kidneys of db/db mice compared to those of db/m mice (Figure 5E). However, C5aRA administration suppressed the up‐regulation of these genes in the kidneys of db/db mice (Figure 5E).

### 3.6 Effects of the C5a/C5aR axis and SCFAs on the STAT3 pathway in HRGECs

**FIGURE 6:**
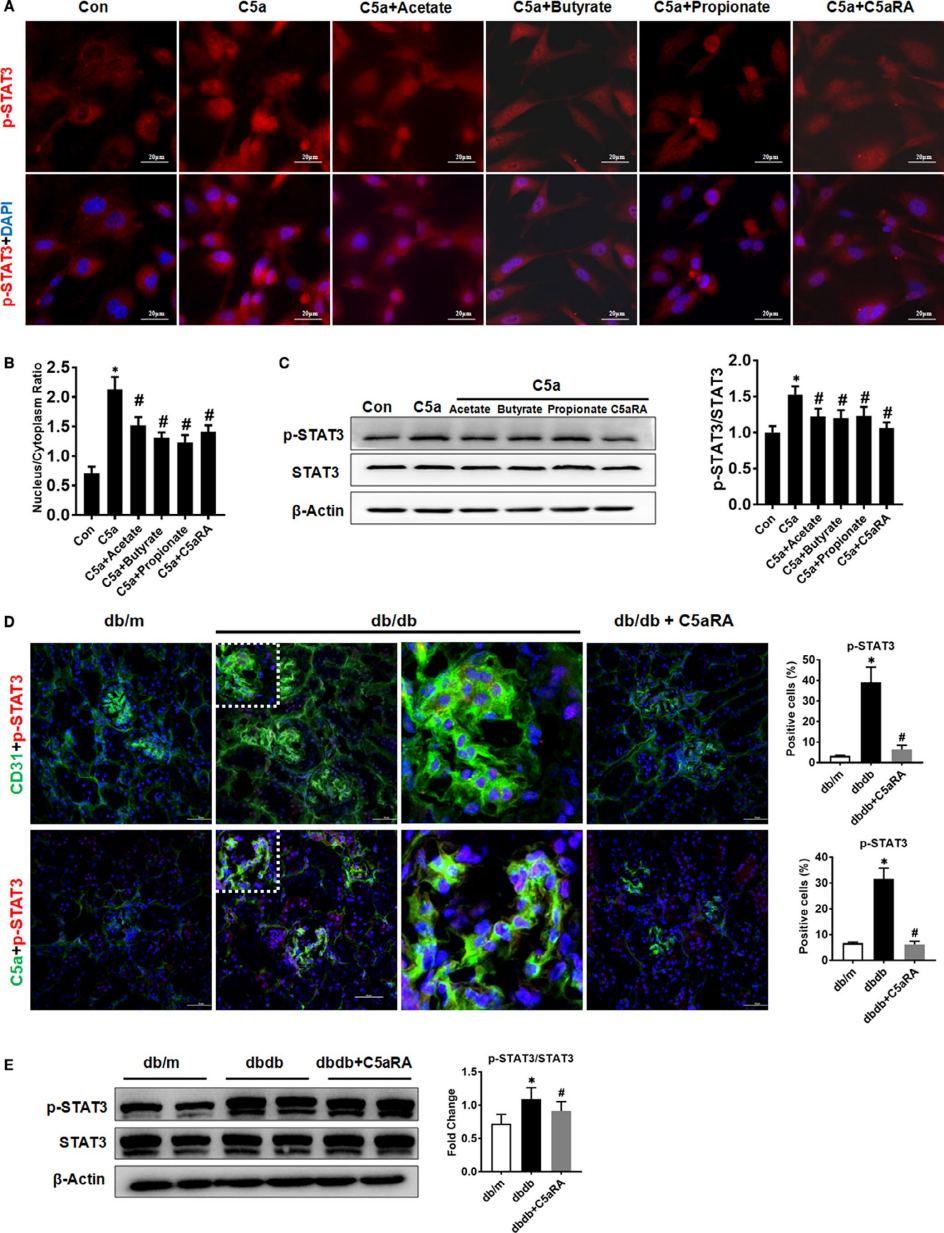
Effect of SCFAs on the C5a/C5aR‐STAT3 pathway in HRGECs. (A) Double‐IF staining of p‐STAT3 in HRGECs after different treatments as indicated (scale bar = 20 µm). (B) Quantification of the nucleus/cytoplasm ratio of p‐STAT3 in HRGECs. (C) Western blot and quantitative analysis of p‐STAT3 and STAT3 protein expression in HRGECs after different treatments as indicated (n = 3, *P < .05, control group vs other groups; ^#^ *P* < .05, C5a + SCFAs or C5a + C5aRA group vs C5a alone group). (D) Double‐IF staining of p‐STAT3 with CD31 or C5a in renal sections from mice (scale bar = 50 µm). (E) Western blotting analysis of p‐STAT3 and STAT3 protein in the renal tissues of mice. *P < .05, db/db or db/db + C5aRA group vs db/m group; ^#^ *P* < .05, db/db + C5aRA group vs db/db group

To reveal the impacts of the C5a/C5aR axis and SCFAs on the STAT3 pathway, HRGECs were stimulated with C5a and treated with SCFAs in vitro. As shown in Figure 6A,B, the levels of nuclear p‐STAT3 were increased in HRGECs under C5a stimulation but could be reduced by SCFA (acetate, butyrate and propionate) or C5aRA treatment. The levels of p‐STAT3/STAT3 were higher in C5a‐stimulated HRGECs than in control HRGECs, but SCFA treatment, similar to C5aRA treatment, suppressed the up‐regulation of p‐STAT3 (Figure 6C). In renal tissues, IF staining showed that p‐STAT3 was up‐regulated in CD31‐positive or C5a‐positive renal GECs but was down‐regulated after C5aRA treatment (Figure 6D). Western blotting confirmed that p‐STAT3 was up‐regulated in the kidneys of db/db mice but was down‐regulated by C5aRA treatment (Figure 6E).

## 4 DISCUSSION

DKD is one of the major causes of morbidity and mortality in diabetic patients, and inappropriate activation of the complement system has been implicated in diabetes and renal diseases.^24^ The plasma levels of C5a are markedly elevated in normoalbuminuric patients with diabetes compared to non‐diabetic controls.^25^ In renal samples from patients with DKD, the complement system has been identified as one of the most significantly regulated pathways,^26^ and activation of the complement system contributes to the progression of DKD.^27^ In line with these reports, we found that C5a was up‐regulated in the sera and kidneys of db/db mice but that the expression of C5aR was not affected by diabetes or C5aR blockade. Endothelial dysfunction strongly contributes to the occurrence and development of DKD.^28^ GECs are the first barriers of the glomerular filtration membrane, and they are vulnerable to damage caused by circulating toxins such as complement C5a.^29^ Interestingly, complement C5a and C5aR were coexpressed in the GECs of kidneys from DKD patients and db/db mice. The glomeruli are the primary sites of diabetic injury in the kidneys, and glomerular hypertrophy is a hallmark of progressive DKD. GECs are key cellular components of the glomerular filtration barrier, and the degree of endothelial dysfunction has been strongly correlated with the severity of DKD.^30^ Therefore, hyperactivation of complement C5 may contribute to the development and progression of diabetic glomerulosclerosis in T2DM. However, other types of renal cells, such as podocytes and tubular epithelial cells, are also involved in the pathology of DKD.^31^, ^32^ Thus, future studies designed to determine whether complement activation also affects other renal cell types and to elucidate the crosstalk between these cells during DKD are needed.

Conversely, complement C5 blockade has been proven to be beneficial in mice with DKD. We have previously found that C5aRA treatment can reduce renal damage in rats with streptozotocin (STZ)‐induced diabetes by inhibiting high glucose‐induced Wnt/β‐catenin signalling and EndMT.^8^ Another study has reported that C5a inhibitor treatment can reduce tubulointerstitial fibrosis by suppressing lipid accumulation and TGF‐β‐driven fibrosis in the kidneys of db/db mice.^33^ In this study, we found that C5aRA treatment improved renal function and attenuated renal fibrosis in db/db mice without affecting glucose or lipid metabolism. Moreover, the proinflammatory cytokine levels and macrophage infiltration in diabetic kidneys were reduced after 4 weeks of C5aRA treatment. These therapeutic outcomes may be because we started the intervention at the early stage of DKD. Based on accumulating evidence, early intervention limits the progression of renal dysfunction and other complications associated with CKD patients.^34^ Similarly, four weeks of empagliflozin treatment was recently shown to attenuate albuminuria and glomerulosclerosis and increased the podocyte filtration slit density in db/db mice.^35^ Thus, early intervention may result in superior therapeutic outcomes by preventing DKD. Interestingly, the C5aRA treatment also reduced the C5a levels in the serum and kidney of db/db mice. Although the exact mechanism is not clear, this effect may be due to the decrease in systemic inflammation, since the complement system is not only an upstream inducer of inflammation but is also regulated by the inflammatory response.^36^ Based on these findings, hyperactivation of C5 in patients with T2DM might promote DKD by inducing inflammatory damage in GECs.

The gut microbiota exerts important trophic and protective effects that are not limited to the gut but can affect the whole body.^37^, ^38^, ^39^ Changes in gut microbiota species richness, diversity or composition may have profound impacts on host physiology by disrupting nutrient utilization and bioactive metabolite synthesis. For example, disruption of gut homeostasis has been associated with obesity, insulin resistance and low‐grade inflammation in humans.^37^ In the context of diabetes, gut dysbiosis may increase gut permeability and facilitate the translocation of toxic products from the gut lumen to the blood, thus potentiating systemic low‐grade inflammation.^38^, ^39^ Specifically, the C5a‐C5aR axis activates the ERK pathway and the production of cytokines such as IL‐8, thereby increasing the permeability of monolayers of intestinal epithelial cells.^40^ In contrast, restoration of the gut microbiome through intermittent fasting has been found to prevent diabetic retinopathy and prolong survival in db/db mice.^41^ In this study, we found that hyperactivation of complement C5 exerted adverse effects on the gut microbiota in db/db mice but that C5aR blockade partly reversed these effects. Moreover, the expression of claudin‐1, an essential protein that maintains the epithelial tight junction and gut permeability, was reduced in subjects with diabetes and partially restored by the C5aRA treatment. Thus, the C5aRA may alter the intestinal inflammatory milieu, thereby affecting the gut permeability and gut microbiota. However, the change in the abundance of certain components of the gut microbiota, such as Proteobacteria, differs from a previous report.^42^ The possible explanation for this discrepancy is that the gut microbiome composition and diversity are affected by multiple genetic and environmental factors, such as species, age, gender, stress and diet, and may even vary in the same individual during different disease states. For instance, an increased abundance of Roseburia has been observed in patients with CKD,^43^ while a reduced abundance Roseburia was also detected in patients with inflammatory bowel disease.^44^ Thus, the detailed mechanism by which activation of the C5a‐C5aR axis impairs gut microbiota function needs to be revealed in future studies.

A previous study has proposed a potential link between the gut and the kidneys, and several gut‐derived factors are thought to affect renal function.^45^ SCFAs, such as acetate, propionate, butyrate, pentanoic acid and isobutyric acid, are fermentation end‐products of dietary polysaccharides produced by the gut microbiota, and declines in the levels of SCFA‐producing saccharolytic microbes are hallmark features of gut dysbiosis.^37^ SCFAs can activate G protein‐coupled receptors expressed on the surfaces of multiple cell types. It is increasingly being recognized that gut microbiota‐derived SCFAs play important roles in regulating the immune response and inflammation in the host. The association of renal function with endogenous SCFAs in DKD is underscored by evidence that the levels of SCFAs are reduced in DKD patients with reduced glomerular filtration rates (GFRs).^45^ Similarly, we found that the levels of gut SCFAs, including acetate, propionate and butyrate, were lower in db/db mice than in controls, which supports the occurrence of gut dysbiosis in T2DM. Notably, SCFA supplementation has been shown to have the potential to attenuate the inflammatory response in a mouse model of acute kidney injury^46^ and in diabetic haemodialysis patients.^47^ In this study, the declines in SCFA levels in db/db mice were partly reversed by C5aR blockade. Therefore, our results indicate that complement C5 contributes to DKD at least partly by inducing gut dysbiosis and lowering gut SCFA production.

**FIGURE 7:**
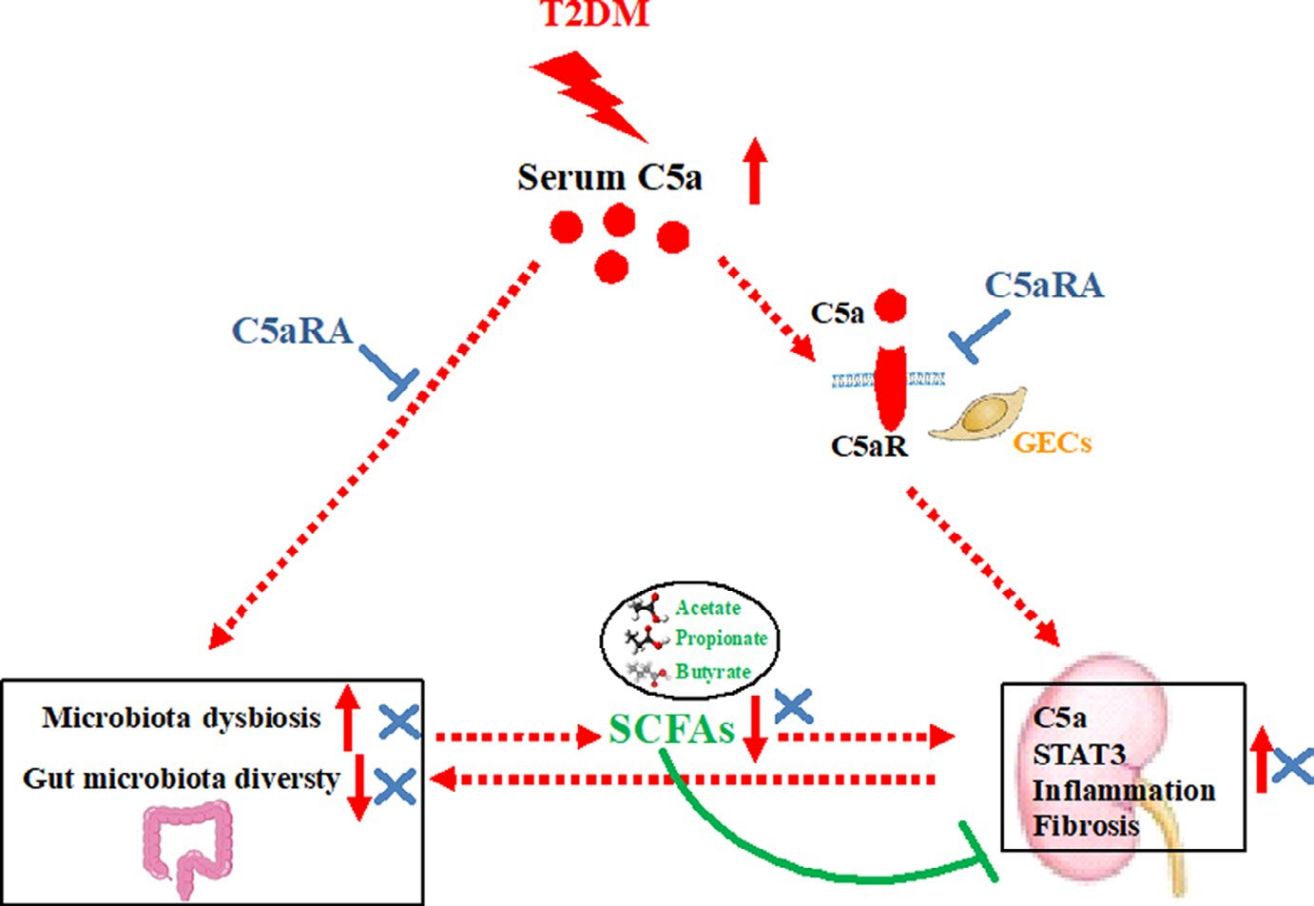
The potential mechanism of complement C5 activation‐induced renal injury in T2DM. In T2DM status, the up‐regulation of C5a activated the STAT3 pathway and thus promoted inflammatory response and further renal fibrosis in kidneys. Meanwhile, the elevated C5a caused gut microbiota dysbiosis (eg declined gut microbiota diversity) with decreased SCFAs production, which impaired the anti‐inflammatory roles of endogenous SCFAs. Conversely, C5aR blockade could attenuate the renal dysfunction through suppressing the STAT3 pathway and restoring the gut‐kidney axis

To further reveal the possible mechanism related to complement C5‐induced renal injury, whole‐genome changes in renal tissues were examined. Interestingly, the majority of pathways affected by complement C5 were linked to innate immune disorders and inflammation. Multiple enriched pathways, such as JAK‐STAT signalling,^48^ NOD‐like receptor signalling,^49^ C‐type lectin receptor signalling^50^ and adipocytokine signalling,^51^ are involved in the mechanisms regulating innate immunity and inflammatory diseases, including T2DM. Specifically, the up‐regulation of the STAT3 pathway, IFN‐I responses and ISGs in the kidneys of db/db mice was suppressed by C5aRA blockade. STAT3 belongs to the STAT family, a class of transcription factors bearing SH2 domains that become activated upon tyrosine phosphorylation and play important roles in regulating the immune response and inflammation.^52^ Many risk factors of diabetes, such as hyperglycaemia and high Ang II levels, can induce cell growth and collagen IV synthesis in glomerular mesangial cells by activating STAT3.^53^, ^54^ In glomeruli isolated from diabetic mice, elevated STAT3 activity is associated with up‐regulation of cytokine expression and extracellular matrix (ECM) synthesis at an early stage of DKD.^55^ GECs are the first barriers of the glomerular filtration membrane, and they are vulnerable to damage by circulating toxins such as C5a. It is believed that GEC dysfunction strongly contributes to the occurrence and development of DKD.^28^ Thus, we examined the impacts of complement C5 and SCFAs on the STAT3 pathway in renal GECs. Indeed, we found up‐regulation of STAT3 in HRGECs with C5a stimulation and in the kidneys of db/db mice. Similarly, C5a has been reported to induce phosphorylation of STAT3 in inflamed rat lungs.^56^ Conversely, we found that SCFA treatment, similar to C5aR blockade, partly reduced the C5a‐induced STAT3 activation in HRGECs. Taken together, complement C5 promotes DKD not only by activating the STAT3 pathway and inflammation in GECs but also by disrupting the gut‐kidney axis in individuals with T2DM (Figure 7).

## 5 CONCLUSION

In summary, hyperactivation of C5a during T2DM led to up‐regulation of STAT3 and the inflammatory response in the GECs of diabetic kidneys. In addition, C5 activation caused gut dysbiosis with limited endogenous SCFA production in db/db mice. In contrast, complement C5 blockade attenuated renal inflammation and dysfunction in db/db mice by suppressing the STAT3 pathway and partly restoring the gut‐kidney axis. The findings of this study suggest that complement C5 is a potential target for the treatment of DKD in T2DM.

## CONFLICT OF INTEREST

The authors report no conflicts of interest in this work.

## AUTHOR CONTRIBUTIONS

**Ling LI:** Data curation (equal); Funding acquisition (supporting); Methodology (equal); Project administration (lead); Resources (equal); Writing‐original draft (lead). **Tiantian Wei:** Data curation (equal); Methodology (equal). **Shuyun Liu:** Data curation (equal); Methodology (equal). **Chengshi wang:** Methodology (equal). **Meng Zhao:** Methodology (equal). **Yanhuan Feng:** Methodology (equal). **Liang Ma:** Methodology (equal); Resources (equal). **Yanrong Lu:** Project administration (equal); Resources (equal). **Ping Fu:** Investigation (equal); Project administration (lead); Resources (equal); Writing‐review & editing (lead). **Jingping Liu:** Methodology (lead); Project administration (lead); Resources (lead); Writing‐review & editing (lead).

## Supporting information

Supplementary Material

## Data Availability

The data that support the findings of this study are available from the corresponding author upon reasonable request.
