# Supplementary material for: Complement C5 activation promotes type 2 diabetic kidney disease via activating STAT3 pathway and disrupting the gut‐kidney axis
Source: J Cell Mol Med. 2020 Dec 6;25(2):960–74. doi: 10.1111/jcmm.16157 (PMC7812276; doi:10.1111/jcmm.16157)
Supplement: Supplementary file 1 — Supplementary Material [file JCMM-25-960-s001.doc]

**Supplemental materials**

**Complement C5 activation promotes type 2 diabetic kidney disease via activating STAT3 pathway and disrupting the gut-kidney axis**

Ling Li^1^, Tiantian Wei^1^, Shuyun Liu^2^, Chengshi Wang^2^, Meng Zhao^2^, Yanhuan Feng^1^, Liang Ma^1^, Yanrong Lu^2^, Ping Fu^1^*, Jingping Liu^2^*

^1^ Kidney Research Laboratory, Division of Nephrology and National Clinical Research Center for Geriatrics, West China Hospital of Sichuan University, Chengdu 610041, China. E-mail: fupinghx@163.com; Fax: +86-28-85423341; Tel: +86-28-85423341

^2^ Key Laboratory of Transplant Engineering and Immunology, Frontiers Science Center for Disease-related Molecular Network, West China Hospital of Sichuan University, Chengdu 610041, China. E-mail: liujingping@scu.edu.cn; Fax: +86-28-85164030; Tel: +86-28-85164029

* Corresponding authors

**
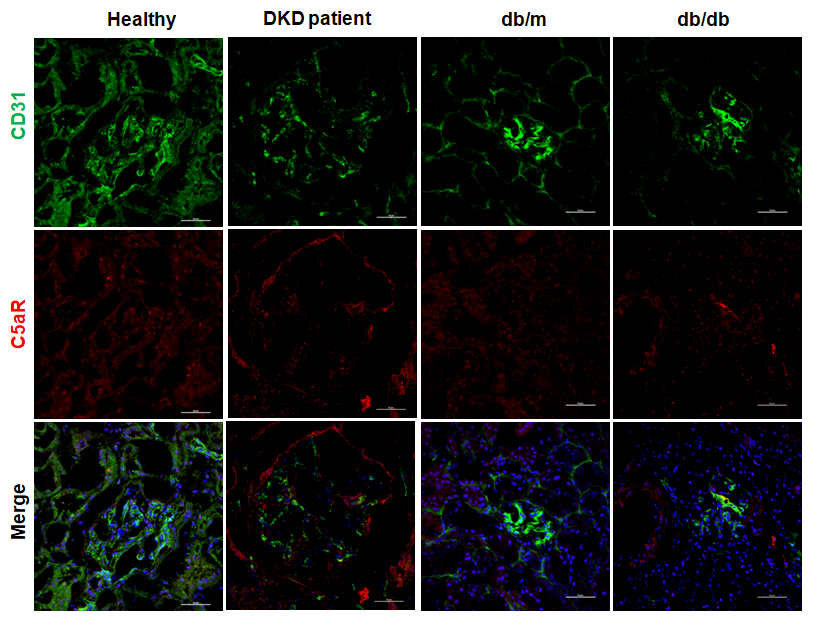
**

**Figure S1.** Double-IF staining of C5a and CD31 in the renal tissues from humans and mice (scale bar = 50 μm).


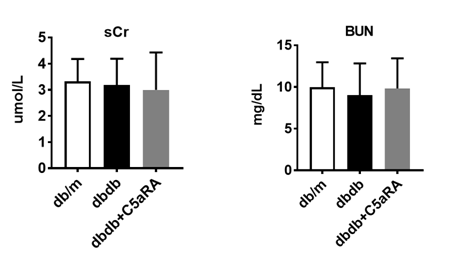


**Figure S2.** Measurement of the serum level of sCr and BUN in mice using clinical biochemistry.

**
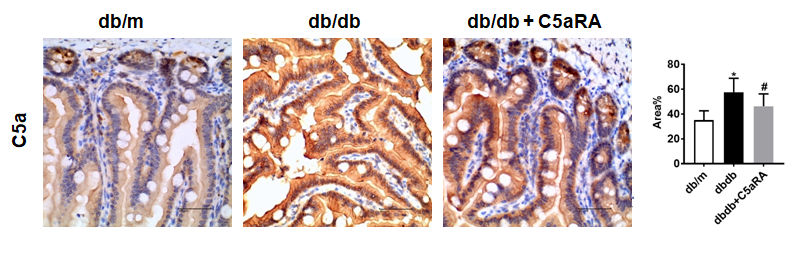
**

**Figure S3.** Measurement of the C5a expression in the intestinal tissues of mice by IHC staining (scale bar = 40 μm).


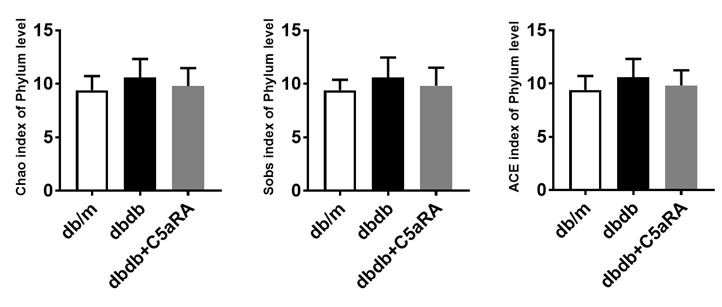


**Figure S4.** Measurement of the sobs, chao, and ace index in gut microbiota of mice by 16S rRNA sequencing analysis.


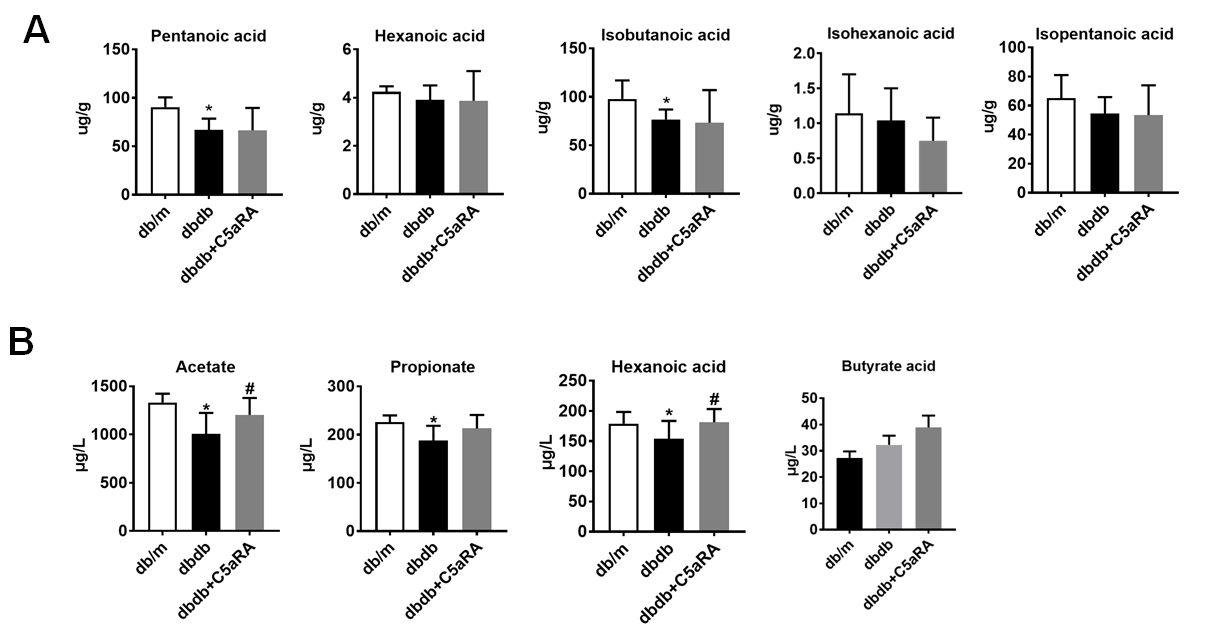


**Figure S5.** (A) The concentration of SCFAs (pentanoic acid, hexanoic acid, isobutanoic acid, isopentanoic acid, and isohexanoic acid) in fecal samples of mice detected by GC-MS. (B) The concentration of SCFAs (acetate, propionate, hexanoic acid, and butyrate acid) in serum samples of mice (* p < 0.05, db/db or db/db + C5aRA group *vs.* db/m group).
